# The Multigene Family Genes-Encoded Proteins of African Swine Fever Virus: Roles in Evolution, Cell Tropism, Immune Evasion, and Pathogenesis

**DOI:** 10.3390/v17060865

**Published:** 2025-06-19

**Authors:** Ruojia Huang, Rui Luo, Jing Lan, Zhanhao Lu, Hua-Ji Qiu, Tao Wang, Yuan Sun

**Affiliations:** State Key Laboratory for Animal Disease Control and Prevention, National African Swine Fever Para-Reference Laboratory, National High Containment Facilities for Animal Diseases Control and Prevention, Harbin Veterinary Research Institute, Chinese Academy of Agricultural Sciences, Harbin 150069, China; huangruojia2024@163.com (R.H.); luorui20210423@163.com (R.L.); lanjing121@163.com (J.L.); luzhanhao106@163.com (Z.L.); qiuhuaji@caas.cn (H.-J.Q.)

**Keywords:** African swine fever virus, multigene family genes, virulence-associated factors, immunomodulatory factors, cell tropism, immune evasion, vaccines, pathogenesis

## Abstract

African swine fever virus (ASFV), the causative agent of African swine fever (ASF), poses a catastrophic threat to global swine industries through its capacity for immune subversion and rapid evolution. Multigene family genes (MGFs)-encoded proteins serve as molecular hubs governing viral evolution, immune evasion, cell tropism, and disease pathogenesis. This review synthesizes structural and functional evidence demonstrating that MGFs-encoded proteins suppress both interferon signaling and inflammasome activation, while their genomic plasticity in variable terminal regions drives strain diversification and adaptation. Translationally, targeted deletion of immunomodulatory MGFs enables the rational design of live attenuated vaccines that improve protective efficacy while minimizing residual virulence. Moreover, hypervariable MGFs provide strain-specific signatures for PCR-based diagnostics and phylogeographic tracking, directly addressing outbreak surveillance challenges. By unifying virology with translational innovation, this review establishes MGFs as priority targets for next-generation ASF countermeasures.

## 1. Introduction

**Figure 1:**
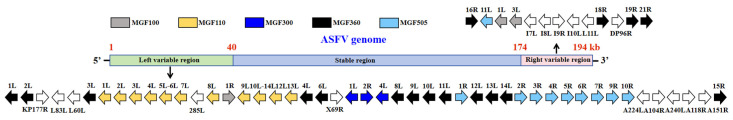
Schematic representation of African swine fever virus (ASFV) multigene family genes (MGFs) located in the left and right variable regions of the viral genome.

African swine fever (ASF), a devastating hemorrhagic disease caused by African swine fever virus (ASFV), has evolved into a global swine industry crisis with the emergence of novel recombinant strains [1]. Since 2023, highly virulent genotype I/II recombinant ASFVs, containing genomic mosaics of historically distinct genotypes, have been detected in China, Vietnam, and the Russian Federation, exhibiting unprecedented lethality and transmissibility in pigs [2,3]. These recombinants evade the immunity induced by genotype II-based live attenuated vaccines (LAVs), posing severe challenges to disease control and global food security [2]. ASFV possesses a large double-stranded DNA genome (170 to 194 kb) divided into three regions: a conserved central region (CCR) flanked by the left variable region (LVR) and the right variable region (RVR) enriched in multigene family genes (MGFs) [4,5] (Figure 1). These MGFs drive genomic diversity and functional adaptability. While non-essential for viral replication in primary porcine alveolar macrophages (PAMs), MGFs critically modulate virulence, immune evasion, and cell tropism [6,7,8,9]. This review comprehensively elucidates that multifunctional MGFs suppress antiviral interferon responses and inflammasome activation, exploit genomic plasticity for adaptive diversification, and offer translational utility in designing LAVs, strain-discriminatory diagnostics, and molecular surveillance tools, thereby positioning MGFs as pivotal targets for advanced ASF control strategies.

## 2. Overview of the MGFs

Since their discovery in the 1980s during studies of ASFV’s genetic diversity [10], MGFs have been identified, with their initial recognition occurring accidentally in the early 1990s through repetitive sequence analysis. The first identified MGFs, *MGF110*, was characterized by Almendral et al. as a highly conserved genes across ASFV strains, playing an essential role in viral infections [11]. In parallel studies, González et al. reported the identification of *MGF360*, which localizes in the LVR of the viral genome and displays significant strain-specific variability, thereby emphasizing the contribution of these genomic regions to ASFV’s genetic plasticity [12] (Figure 1). Subsequent research by Vydelingum et al. revealed that *MGF100*, positioned at the RVR, is involved in immune evasion [13] (Figure 1). Further investigations led to the discovery of *MGF530* (later reclassified as *MGF505*) and *MGF300*, which exhibit unique protein dimensions and functional properties [14,15]. The identified MGFs account for approximately 30% of the entire ASFV genome. In addition to that, the study classified the MGFs-encoded proteins into 31 groups based on protein sequence similarity and network clustering to provide a foundation for further research on their roles in viral infection and pathogenesis [16]. Member numbers documented to date vary across MGFs: *MGF100* contains three members, *MGF110* contains fourteen, *MGF300* contains three, *MGF360* contains twenty-two, and *MGF505* contains eleven [17,18]. It is important to note that the specific numbers of MGFs can be gained or lost in different ASFV strains.

ASFV has evolved into both virulent and avirulent strains through genetic evolution, with the hallmark of avirulent strains being the significant deletion of MGFs regions—genetic elements recognized as critical virulence-associated factors. This genomic deletion strongly implies that MGFs regulate ASFV pathogenicity, thereby positioning them as prime targets for rationally designed LAVs [19]. Notably, during ASFV adaptation to immortalized cell lines (e.g., HEK293T and Vero cells) [20,21,22,23], MGFs deletions were found to concurrently decrease viral replication in PAMs, revealing their role in modulating viral adaptation [8,24,25,26,27]. Meanwhile, the emergence of highly pathogenic genotype I/II recombinant ASFV strains has substantially complicated ASFV containment strategies, necessitating advanced tools for strain differentiation and surveillance. In this context, MGFs offer unique diagnostic advantages due to their genetic diversity: specifically, their polymorphic nature enables precise strain differentiation while supporting geographic tracing capabilities, thereby serving as critical markers for molecular epidemiology [28,29,30,31].

## 3. The Driving Factors of Diversity During the ASFV Pandemic

Since the initial identification of ASFV in 1921, its global dissemination and evolutionary adaptation have led to substantial genetic diversity, with at least 24 genotypes and 9 serogroups identified [32]. Over the past century, ASFV have exhibited significant variations in virulence, attributed to genetic changes such as point mutations, insertions, deletions, and large genomic deletions. These alterations disrupt gene expression through premature termination, frameshifts, or modifications to amino acid sequences [33]. Comparative genomic analyses highlight MGFs, particularly *MGF110* and *MGF360*, as central drivers of variability. For instance, attenuated ASFV strains like NH/P68 and OURT88/3, when compared to the virulent Lisbon 60 (L60) strain, show extensive deletions in *MGF110* (*-5L*, *-6L*, *-7L*, *-11L,* and *-12L*), and *MGF360-6L* genes, alongside insertions of *MGF110* (*-4L*, *-5L*, and *-9L*) and *MGF100-1R* genes [33]. Structural plasticity in MGFs is further evidenced by gene splitting events, such as the *MGF110-2L/13L* fusion gene in the L60 strain dividing into *MGF110* (*-2L* and *-14L*) genes in the NH/P68 strain, and inversions like those observed in the Estonia 2014 strain, which lost most *MGF110* except *MGF110-14L* gene and duplicated a genomic segment from *MGF110-11L* gene to *DP60R* gene [33]. Geographical and temporal factors also shape diversity: ASFV strains circulating in Portugal (NH/P68 and OURT88/3) maintain conserved deletion patterns over decades, whereas Chinese strains (HuB20) display distinct genetic profiles [33]. The NH/P68 strain, isolated eight years after L60, shares 99.65% genomic similarity but carries additional *MGF100-1R* and *MGF110* (*-4L*, *-5L*, and *-9L*) genes and a 4458-nucleotide insertion between the *MGF110-2L* and *MGF110-13L* genes, likely contributing to its attenuated phenotype [34].

Parallel to natural evolution, in vitro studies using primary porcine monocytes and PAMs face challenges, such as ethical concerns, high variability, and poor reproducibility [35], driving the need for immortalized cell lines. Laboratory cell-adapted ASFV strains demonstrate characteristic MGFs alterations: Vero-adapted strains (L60V [20], BA71V [21], and ASFV-G/V [23]), HEK293T-adapted [22], and PIPEC-adapted ASFV strains [36] consistently exhibit deletions in both LVR and RVR. Notably, an MA104-adapted ASFV strain shows an extensive deletion spanning *MGF100* to *MGF505* [37], while COS7-adapted ASFV strains (Lv17/WB/Rie1 and Estonia) display distinctive 5′ terminal duplications [38]. This genomic plasticity—manifesting as deletions, insertions, inversions, and duplications—underpins ASFV diversity. Such variations emerge through selective pressures during host invasion, geographical expansion, and laboratory adaptation, effectively linking natural and experimental evolutionary trajectories.

The observed MGFs deletions during ASFV adaptation significantly impact viral virulence and phenotypic characteristics. These findings have critical implications for (1) developing targeted control measures, particularly LAVs design; (2) advancing fundamental understanding of MGFs functional roles; (3) elucidating the molecular mechanisms governing ASFV genetic plasticity. Continued investigation in these areas will enhance our capacity to develop next-generation vaccines and refined diagnostic approaches against ASFV.

## 4. The Diversity and Significance of the MGFs-Encoded Proteins

### 4.1. Immunomodulatory Factors: Regulating IFNs and Inflammatory Responses

MGFs-encoded proteins of ASFV are key modulators of host innate immune responses. MGFs-encoded proteins interfere with multiple signaling cascades, including the cGAS-STING, JAK-STAT, and NF-*κ*B signaling pathways, suppressing the production of the type I interferons (IFNs) and pro-inflammatory cytokines. Such immunomodulatory strategies contribute significantly to viral immune evasion and replication.

**Figure 2:**
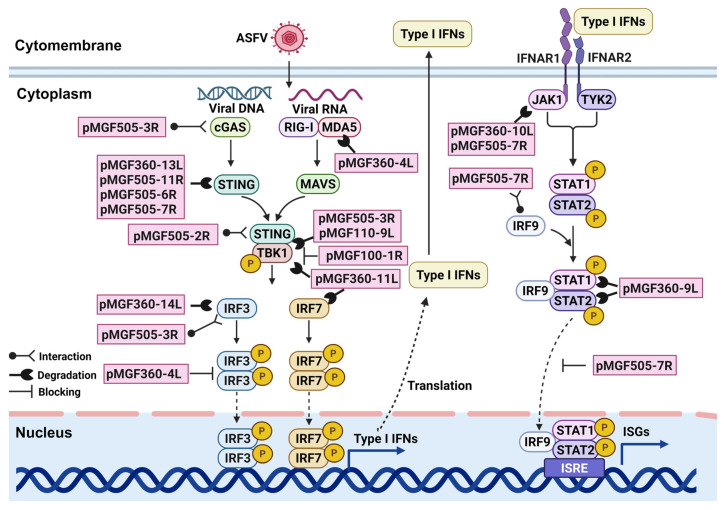
The proteins encoded by multigene family genes (MGFs) involved in the type I IFNs signaling pathway. The pink box indicates the MGFs-encoded proteins.

The cGAS-STING signaling pathway is essential for sensing cytoplasmic viral DNA and initiating the production of type I IFNs. Upon recognition of viral DNA, cGAS catalyzes the synthesis of cyclic GMP-AMP (cGAMP), which activates the adaptor protein stimulator of interferon genes (STING) [39,40]. The activated STING translocates from the endoplasmic reticulum to the Golgi apparatus, where it recruits and activates TANK-binding kinase 1 (TBK1), leading to the phosphorylation and nuclear translocation of interferon regulatory factor 3 (IRF3) [41]. IRF3 subsequently induces transcription of the type I IFNs and other antiviral genes [41]. Several MGFs-encoded proteins target this pathway at different levels. For instance, pMGF505-6R promotes STING degradation via the autophagy pathway [42], while pMGF505-11R mediates STING degradation through the lysosome, ubiquitin–proteasome, and autophagy pathways [43]. The downstream pMGF505-3R facilitates the degradation of TBK1 via the autophagy pathway [44]. Moreover, pMGF360-4L interacts with IRF3, inhibiting its phosphorylation and thereby reducing the type I IFNs transcriptions [45] (Figure 2) (Table 1).

The JAK-STAT signaling pathway is critical for transducing IFNs signals and activating interferon-stimulated genes (ISGs). Following IFN binding to their cognate receptors (IFNAR1 and IFNAR2), the associated Janus kinase 1 (JAK1) and Tyrosine kinase 2 (TYK2) undergo reciprocal phosphorylation, subsequently activating signal transducer and activator of transcription 1 (STAT1) and signal transducer and activator of transcription 2 (STAT2) [66]. These transcription factors dimerize and form the ISGF3 complex with interferon regulatory factor (IRF9), which translocates to the nucleus to initiate ISGs transcription [67]. ASFV inhibits this pathway through the degradation of adaptor proteins by MGFs-encoded proteins. For example, both pMGF360-10L and pMGF505-7R target JAK1 for degradation via ubiquitin–proteasome and autophagy pathways, respectively [25,63]. pMGF360-9L promotes the degradation of STAT1 via the apoptotic pathway and STAT2 via the ubiquitin–proteasome pathway [51]. This effectively impairs ISGF3 formation and downstream ISGs expression, limiting the establishment of an antiviral state (Figure 2) (Table 1).

**Figure 3:**
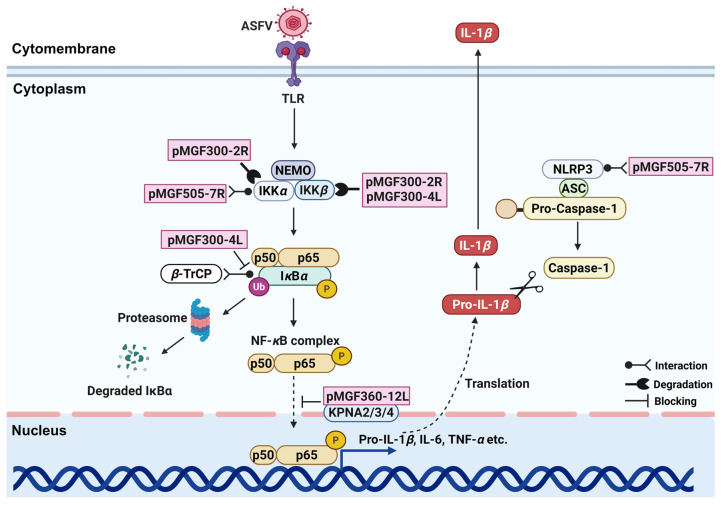
The proteins encoded by multigene family genes (MGFs) involved in the NF-*κ*B signaling pathway. The pink box indicates the MGFs-encoded proteins.

The activation of the NF-*κ*B signaling pathway is crucial for producing pro-inflammatory cytokines, such as TNF-*α* and IL-1*β*. Upon receptor stimulation (TNFR1 and TNFR2), the IKK complex phosphorylates I*κ*B*α*, leading to its degradation and subsequent nuclear translocation of NF-*κ*B dimers (p65 and p50). ASFV utilizes multiple mechanisms to suppress this signaling axis. Our study identified that pMGF300-2R promotes autophagy-lysosomal degradation of IKK*α* and IKK*β*, inhibiting I*κ*B*α* phosphorylation [27]. Additionally, pMGF300-4L competitively binds I*κ*B*α*, interfering with *β*-TrCP-mediated ubiquitination and degradation of IκBα [26] (Figure 3) (Table 1).

In summary, MGFs-encoded proteins play a central role in the regulation of ASFV infectivity and pathogenicity by targeting pivotal components of the cGAS-STING, JAK-STAT, and NF-*κ*B signaling pathways. Through diverse mechanisms such as proteasomal or autophagy-mediated degradation, inhibition of phosphorylation events, and disruption of nuclear translocation, these MGFs-encoded proteins effectively inhibit the production of the IFNs and pro-inflammatory cytokines, thereby facilitating immune evasion and establishing a favorable environment for ASFV replication.

### 4.2. Important Virulence-Associated Factors: Regulation of ASFV Infectivity and Pathogenicity

MGFs are essential in regulating ASFV infectivity and pathogenicity. Deletions of MGFs, whether single or combined, reduce the virulence of the ASFV. Accordingly, the simultaneous deletion of *MGF360* (*-12L*, *-13L*, and *-14L*) and *MGF505* (*-1R*, *-2R*, and *-3R*) genes from the virulent ASFV Georgia isolate (ASFV-G-ΔMGF) resulted in the complete loss of pathogenicity in pigs [68]. The pigs inoculated with 10^2^ or 10^4^ HAD_50_ of the ASFV-G-ΔMGF strain remained clinically healthy throughout the 21-day observation period, although viremia persisted for a prolonged duration. After 21 days, a challenge with the parental ASFV strain led to generally normal clinical outcomes, demonstrating that the deletions had effectively attenuated the ASFV [68]. The same six MGFs gene-deleted mutant, HLJ/18-6GD, was also fully attenuated and provided complete protection against a lethal challenge with the parental virulent virus [69]. However, it has been reported that the HLJ/18-6GD vaccine candidate reverted to virulence after six back-passages in pigs, although it remains unclear how this reversal occurred [69]. In a similar study, the simultaneous deletion of ten *MGF360* (-*9L*, -*10L*, -*11L*, -*12L*, *-13L*, and -*14L*) and *MGF505* (-*1R*, -*2R*, -*3R*, and *-4R*) genes from the genotype I Benin 97/1 ASFV strain (Benin-ΔMGF) resulted in a significant attenuation of the virus virulence in pigs. Pigs inoculated with the Benin-ΔMGF deletion mutant virus (10^2^ TCID_50_) exhibited markedly reduced virulence compared with those infected with the virulent Benin 97/1 strain. Following a challenge with the virulent Benin 97/1 strain, all five pigs survived without showing clinical signs [70]. Interestingly, the same ten MGFs gene-deleted mutant (Georgia-ΔMGF) attenuated the virus virulence in pigs [8]. Pigs were immunized with 10^4^ HAD_50_ of the Georgia ΔMGF and subsequently boosted with the same dose 19 days after the initial immunization. However, only 25% of the pigs that received a two-dose intramuscular immunization with Georgia-ΔMGF were protected against a lethal challenge with the parental virulent virus [8]. Thus, despite having the same MGFs deletions, the level of protection conferred by Georgia-ΔMGF was significantly lower than that provided by Benin-ΔMGF.

The pigs infected with ASFV-ΔMGF110/360-9L, a mutant with deletions in the *MGF110*-*9L* and *MGF360*-*9L* genes, showed significantly milder clinical signs than those infected with the parental CN/GS/2018 strain. The ASFV-ΔMGF110/360-9L group survived in the 17-day observation period, with only a brief fever and no severe symptoms. Pathological analysis revealed less extensive organ damage, and the ASFV-ΔMGF110/360-9L-infected pigs had lower viral loads in blood, oral, nasal, and stool swabs. Additionally, these pigs exhibited higher levels of ASFV-specific antibodies, indicating a robust immune response [71]. When later challenged with the CN/GS/2018 strain, these pigs remained healthy. Similarly, pigs inoculated with the ASFV-Δ9L/Δ7R (a virus with deletions in the *MGF360*-*9L* and *MGF505*-*7R* genes) showed significantly reduced virulence compared with those infected with the parental CN/GS/2018 strain. The ASFV-Δ9L/Δ7R-infected pigs group remained clinically healthy throughout the observation period, with no severe symptoms or complications. Pathological analysis revealed minimal organ damage in the ASFV-Δ9L/Δ7R group, with lower viral loads and reduced viral shedding, suggesting that ASFV-Δ9L/Δ7R is attenuated and provides 83.3% protection against a lethal homologous ASFV challenge [62].

Genetic deletion of specific MGFs in ASFV strains consistently demonstrates significant viral attenuation. The *MGF110-9L*, *MGF360-9L*, *MGF505-7R*, *MGF300-2R*, and *MGF300-4L* (in both the CN/GS/2018 and HLJ/18 strains) deletions all showed markedly improved survival rates (50–100% vs. parental strain mortality), reduced clinical symptoms, and significantly lower viral loads in blood and tissues [24,26,27,51,63,72]. Particularly notable was the *MGF505-7R* deletion in the CN/GS/2018 strain (10 HAD_50_) and the *MGF505-2R* deletion in the Arm/07 strain (10^2^ TCID_50_), achieving 100% survival versus the parental strain’s 100% mortality within 15 days [60,72]. The *MGF505-2R* deletion in the Arm/07 strain (10^2^ TCID_50_) vaccinated pigs challenged with the virulent ASFV Korean isolate ASF/Korea/Pig/Paju/2019 showed minimal viral replication and a 75% survival rate. In a prime-boost regimen (both 10^3^ TCID_50_), upon the parental Arm/07 strain challenge, vaccinated pigs exhibited a 60% survival rate, with two non-survivors linked to bacterial coinfection [60].

These findings collectively reveal that targeted MGFs deletions create effective LAVs candidates, with efficacy varying by specific gene deletion, viral strain, and inoculation dose. The consistent attenuation patterns across different MGFs and ASFV strains highlight the crucial role of MGFs in viral pathogenesis and their potential as precise targets for rational vaccine design. Importantly, certain MGFs (including *MGF100-1R*, *MGF110-1L*, *MGF110-5L-6L*, *MGF110-11L*, *MGF360-1L*, *MGF360-16R*, and *MGF360-18R*) were identified as non-essential, showing no impact on in vitro replication or in vivo virulence when deleted [58,73,74,75,76,77,78].

### 4.3. Viral Cell Tropism Determinants

ASFV displays a marked tropism for monocyte–macrophage lineage cells, particularly PAMs, the predominant immune cells in the lung and a key target for viral replication. These cells, commonly used as the primary in vitro model for ASFV studies, are critical for understanding viral pathogenesis. Accumulating evidence has identified MGFs-encoded proteins—especially members of the *MGF360* and *MGF505*—as pivotal determinants of viral cell tropism. Variations or deletions in these MGFs significantly alter the ASFV’s ability to replicate in PAMs, underscoring their role in mediating host cell specificity and shaping the ASFV’s replication profile.

In an investigation into the ASFV’s adaptation to cell lines, it was discovered that the ASFV strains MS16 and BA71V, which were passaged in non-macrophage cell lines, failed to replicate in PAMs [79]. Instead of viral progeny production, these strains caused early cell death in PAMs. Further genomic analysis led to the identification of a crucial region in the ASFV genome, specifically *MGF360* and *MGF505*, as key determinants for PAM tropism. The removal of six genes *MGF360* (-*4L*, -*6L*, -*9L*, -*10L*, and -*11L*) and two *MGF505* (-*1R* and -*2R*) genes from the virulent ASFV Pr4 strain resulted in a marked reduction in viral replication in PAMs [79]. Recent studies have also confirmed this view, with MGFs serving as determinants for host cells. Many studies have also shown that the deletion of a single MGFs or the large-scale deletion of multiple MGFs can impact the replication of ASFV strains in PAMs. For instance, targeted deletion of *MGF360*-*10L* (CN/GS/2018 strain), *MGF300*-*2R*, *MGF300*-*4L* (HLJ/18 strain), *MGF110*-*9L* (HLJ/18 strain), and *MGF360*-*12L* (ASFV Georgia) reduced viral titers by approximately 10-fold compared withparental strains [8,24,25,26,27]. Similarly, the Benin-ΔMGF strain (lacking ten genes of *MGF505/360*) exhibited a 10-fold replication defect in PAMs relative to the wild-type Benin 97/1 isolate [70]. However, deletions of overlapping MGFs subsets (lacking nine genes of *MGF505/360*) in the virulent ASFV Georgia strain showed no replication in PAMs [8,68], suggesting that functional redundancy or strain-specific genetic contexts may compensate for certain MGFs losses. These findings collectively underscore the heterogeneous roles of MGFs in viral adaptation, and all of the above research provides further evidence to suggest that the impact of deleting these MGFs on growth in PAMs may be strain-specific.

Moreover, MGFs are critical for ASFV’s persistence in its biological vectors, *Ornithodoros* ticks. ASFV establishes high-titer, persistent infections in competent *Ornithodoros* species [80]. Studies demonstrate that deletions within the *MGF360* region impair the ASFV’s ability to establish generalized infections in its arthropod vectors, *Ornithodoros porcinus* ticks. The ASFV mutant lacking *MGF360* exhibits severely reduced replication in the midgut and salivary glands of ticks, preventing efficient viral transmission [79,81]. These findings underscore the essential function of MGFs in both the viral adaptation to mammalian and arthropod vectors, contributing to the ASFV’s persistence and spread across both domestic pig and wild boar populations, as well as its tick vectors.

Thus, MGFs are not only critical determinants of ASFV’s tropism for PAMs but also essential for efficient replication and transmission within its arthropod vectors, influencing the overall viral spread and persistence. These findings provide crucial insights into the molecular mechanisms governing ASFV-host interactions and offer valuable information for developing control measures targeting these genetic determinants.

## 5. Translational Applications of MGFs-Related Research

### 5.1. Molecular Markers of Genetic Evolution in ASFV

MGFs of ASFV are crucial for understanding the genetic evolution and molecular epidemiology of the virus. Due to the high variability observed in MGFs across different ASFV strains, they have become essential molecular markers for tracking the ASFV’s transmission and evolution across various regions. Recent studies have highlighted that specific MGFs, such as the *MGF110*-*1L*, *MGF505*-*10R*, and *MGF360*-*21R* genes, exhibit significant sequence variation, providing important insights into intra-epidemic genetic diversity within ASFV populations [28]. These MGFs are highly variable in both wild boar and domestic pig populations, and their genetic divergence serves as a tool for differentiating between geographically distinct ASFV strains. For instance, the *MGF505*-*5R* gene has been identified as a pivotal marker for distinguishing ASFV strains originating from Eastern Europe and Asia, including isolates from Poland and Russia, underscoring its utility in phylogenetic analyses and epidemiological tracing [29]. Of particular importance, mutations within MGFs have been associated with the genetic clusters of ASFV strains, contributing to understanding the virus’s transmission dynamics. Mutations in MGFs such as the *MGF110*-*1L* and *MGF505*-*9R* genes have been recognized as specific mutations that define specific genetic clusters, providing critical data for tracking the spread of ASFV [31]. Similarly, in the Russian Federation, significant sequence diversity has been detected in MGFs such as the *MGF360*-*10L* and *MGF505*-*9R* genes, which enables the differentiation of ASFV isolates from wild boar populations in Eastern and Western regions, further facilitating the identification of ASFV’s geographic origin [30].

Overall, the MGFs, due to their high variability and widespread presence across ASFV strains, are indispensable as molecular markers for studying the genetic evolution, transmission, and epidemiology of ASFV. Their ability to differentiate between strains from different regions and their role in defining genetic clusters makes them invaluable tools for global ASF control efforts, epidemiological surveillance, and the development of diagnostic assays.

### 5.2. Early Detection and Differential Diagnosis Targets

**Figure 4:**
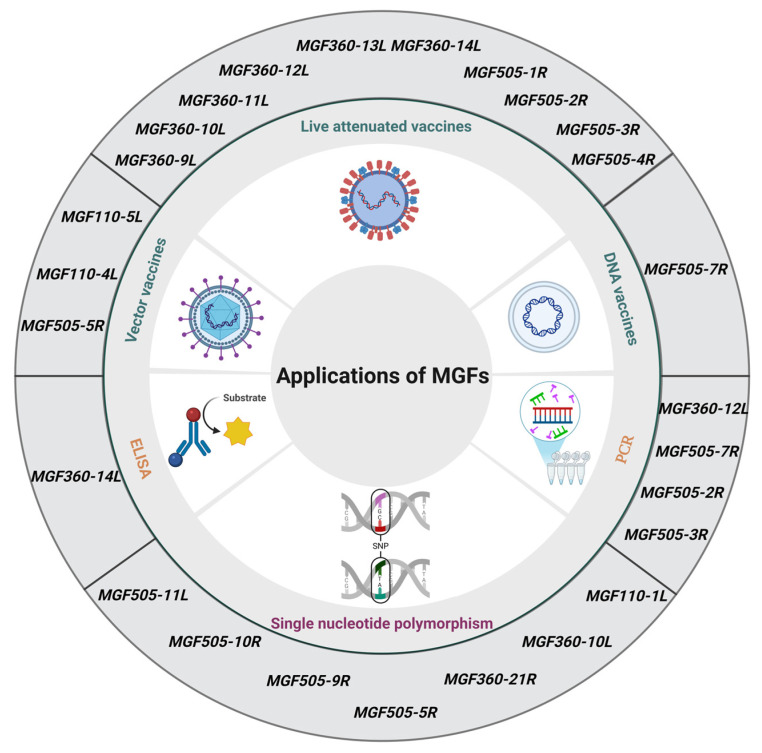
The applications of the proteins encoded by multigene family genes (MGFs).

Due to a lack of effective vaccines or drugs for combating ASFV, the early detection and accurate diagnosis of ASFV are critical for controlling outbreaks and minimizing the economic impact of the disease. Quantitative PCR (qPCR) is recommended by the World Organisation for Animal Health (WOAH) for laboratory diagnosis of ASFV. Currently, qPCR is widely used to detect the ASFV from a very early stage of infection in tissues, ethylene diamine tetra-acetic acid (EDTA)-blood, and serum samples [82]. Due to the widespread presence of ASFV variant strains and the emergence of gene-deleted vaccines, there has been increasing attention on the *EP402R* (*CD2V*) and *I177L* genes and MGFs [82]. Several qPCR assays have been developed to target key genes, including multiple assays targeting the *B646L*, *I177L*, *MGF505*-*2R*, and *EP402R* genes [83], multiple assays targeting the *B646L*, *EP402R*, *MGF505*-*3R*, and *A137R* genes [84], multiple assays targeting the *I177L*, *EP402R*, and *MGF360*-*14L* genes [85], triple assays targeting the *B646L*, *MGF360*-*14L*, and *EP402R* genes [86], triple assays targeting the *MGF360*-*12L*, *UK*, and *I177L* genes [87], dual assays targeting the *B646L* and *MGF505*-*2R* genes [88], assays targeting the *MGF505*-*7R* gene [89]. Additionally, crystal digital PCR (cdPCR), a digital PCR based on microfluidic technology targeting the *B646L*, *MGF505*-*2R*, and *I177L* genes, has been developed [90]. These assays are capable of distinguishing the wild-type ASFV strain from gene-deleted variants. A current study indicates that a duplex fluorescent qPCR assay targeting the *O61R* and *MGF110*-*1L* genes can also distinguish genotype I, genotype II, and genotype I/II recombinant ASFVs in China [91] (Figure 4).

### 5.3. New Targets for Designing Vaccines

As early as the mid-1960s, developing a safe and effective ASF vaccine has already commenced. Various methodologies were explored, including inactivated, subunit, DNA, viral vector, and LAVs [19]. The targeted deletion of virulence-associated genes (VAGs) from virulent strains has become a pivotal strategy in the development of LAVs candidate in current ASF research.

The simultaneous deletion of *MGF505* (*-1R*, *-2R*, and *-3R*), MGF360 (-*12L*, -*13L*, and -*14L*) genes, and the *EP402R* gene from the HLJ/18 strain (HLJ/18-7GD) was fully attenuated. A single-dose intramuscular immunization of young pigs with 10^3^ or 10^5^ TCID_50_ of the HLJ/18-7GD virus protected against lethal challenge with the virulent parental virus [69]. The HLJ/18-7GD has maintained phenotype stability in virulence reversion tests, has completed both laboratory research and clinical trials, and is awaiting the acquisition of a transgenic safety certificate [69]. The simultaneous deletion of the carboxyl-terminal half of the *MGF505* (-*1R*, -*2R*, and -*3R*) and *MGF360* (-*12L*, -*13L*, and -*14L*) genes and the *9GL* gene from the virulent ASFV Georgia strain (ASFV-G-Δ9GL/ΔMGF) was significantly attenuated virulence in swine, as demonstrated by its inability to cause clinical disease even at high doses. Importantly, animals inoculated with various doses (from 10^2^ to 10^6^ HAD_50_) did not show detectable levels of the virus or develop anti-ASFV antibodies during the observation period. Despite these promising findings regarding attenuation, ASFV-G-Δ9GL/ΔMGF did not confer protection against a subsequent challenge with the virulent parental ASFV genotype II Georgia isolate [92]. The simultaneous deletion of *MGF360* (-*12L*, -*13L*, and -*14L*) genes, the *I177L* and *CD2v* genes from the ASFV GZ201801 strain (ASFV-GZΔI177LΔCD2vΔMGF) led to a fully attenuated virus that does not convert into a virulent strain in pigs, thus making it a promising candidate for a live, attenuated ASF vaccine [93]. The vaccinated pigs showed excellent protection against lethal challenges with the parental ASFV GZ201801 strain without showing severe adverse effects like fever, joint swelling, or viremia [93]. Importantly, the vaccine showed high genetic stability, did not induce viral shedding, and provided complete immunity during the challenge period [93] (Figure 4).

In addition to conventional LAVs, subunit vaccines targeting specific antigens from ASFV have attracted interest as a safer and potentially more efficacious approach to immunological protection. The effective commercialization of various subunit vaccines, including those targeting classical swine fever [94], porcine circovirus type 2 [95], and infectious bursal disease virus [96], establishes a robust platform for the advancement of novel vaccines, particularly for ASFV. MGFs are also designed as target antigens for subunit vaccines. Immunization with DNA plasmids encoding ASFV antigens M448R and MGF505-7R, followed by a lethal dose ASFV challenge in vaccinated pigs, resulted in an observed increase in the survival rate of the immunized pigs [97]. A pool consisting of the ASFV proteins A151R, B646L, C129R, CP204L, CP530R, E146L, I73R, I125L, L8L, M448R, MGF110-4L, and MGF110-5L, delivered via recombinant adenoviruses for priming and modified vaccinia Ankara (MVA) for boosting, resulted in a reduction in clinical signs and viremia levels in a subset of pigs following challenge with the virulent genotype I isolate, OUR T1988/1 [98]. Immunization with recombinant adenoviruses, each expressing one of eight ASFV proteins (including B602L, B464L, CP204L, E183L, E199L, EP153R, F317L, and MGF505-5R), followed by a booster dose with MVA expressing the same proteins sufficient to protect pigs from challenge with the virulent genotype I isolate, OUR T1988/1 [99]. Subsequent research further substantiated that immunization with recombinant adenoviruses, each expressing one of six ASFV proteins (including B602L, E183L, E199L, EP153R, F317L, and MGF505-5R) delivered through recombinant adenoviruses prime followed by recombinant adenoviruses boost and recombinant adenoviruses the second boost (including B602L, E183L, EP153R) also can protect pigs from challenge with the virulent genotype I isolate, OURT1988/1 [99] (Figure 4).

## 6. Conclusions and Prospects

The MGFs-encoded proteins of ASFV are fundamental to viral pathogenesis, immune evasion, and cell tropism. Notably, pMGF100-9L, pMGF300-2R pMGF300-4L, pMGF360-9L, and pMGF505-7R are critical virulence determinants that suppress host innate immune responses, including the type I IFNs and NF-*κ*B signaling pathways. Moreover, the MGFs-encoded proteins significantly influence cell tropism and replication efficiency in PAMs, underscoring their importance in host-pathogen interactions.

The deletion of specific MGFs has emerged as a promising strategy for developing LAVs, with several candidates demonstrating attenuation while preserving immunogenicity. For example, deletions in *MGF505* (*-1R*, *-2R*, and *-3R*), *MGF360* (*-12L*, *-13L*, and *-14L*) genes in strains results in ASFV-G-ΔMGF and HLJ/18-7GD able to protect against lethal challenges, presenting a pathway toward effective vaccine development [68,69]. Nonetheless, strain-specific differences in the MGFs-encoded protein’s functionality and the risk of virulence reversion highlight the necessity for rigorous assessments of genetic stability and tailored approaches for diverse ASFV genotypes.

Future research should prioritize elucidating the structural and mechanistic details of the MGFs-encoded proteins to identify conserved targets for treatment. Advances in subunit vaccines utilizing immunogenic the MGFs-encoded proteins could complement LAVs strategies. Furthermore, integrating genomic surveillance of MGFs dynamics into outbreak management will enhance our ability to monitor viral evolution and recombination, particularly in regions where genotype I/II recombinant ASFVs are circulating. Innovations in diagnostics, such as multiplex qPCR targeting MGFs, will improve the differential detection of wild-type and vaccine strains.

In conclusion, MGFs present both challenges and opportunities in ASF control. A multidisciplinary approach incorporating molecular virology, structural biology, and immunology will be essential to leverage these insights for developing next-generation vaccines and antiviral strategies, ultimately mitigating the global impact of ASF.

## Figures and Tables

**Table 1:** The functions of the MGFs-encoded proteins.

Multigene Family	Members	Functions	References
*MGF100*	*1L*	Unknown	/
	*1R*	Blocks the dimerization of TBK1	[46]
	*3L*	Unknown	/
*MGF110*	*1L*	Unknown	/
	*2L*	Unknown	/
	*3L*	Unknown	/
	*4L*	Unknown	/
	*5L* *–6L*	Unknown	/
	*7L*	Induces translational repression and ISGs formation in host cells by activating the PERK/PKR-eIF2*α* pathway	[47]
	*8L*	Unknown	/
	***9L***	Facilitates TBK1 degradation by upregulating PIK3C2B	[48]
	*10L* *–14L*	Unknown	/
	*12L*	Unknown	/
	*13L*	Unknown	/
*MGF300*	*1L*	Unknown	/
	***2R***	Promotes K27-linked polyubiquitination of IKK*α* and IKK*β* by recruiting the cargo receptor TOLLIP for selective autophagic degradation; Promotes IKK*β* ubiquitination by recruiting the E3 ubiquitin ligase TRIM21	[27,49]
	***4L***	Promotes the autophagic degradation of IKK*β* and increasing the stability of I*κ*B*α*	[26]
*MGF360*	*1L*	Unknown	/
	*2L*	Unknown	/
	*3L*	Unknown	/
	*4L*	Inhibits IRF3 phosphorylation; Interacts with MDA5 and recruits the mitochondrial selective autophagy receptor SQSTM1 (p62), leading to the degradation of MDA5	[45,50]
	*6L*	Unknown	/
	*8L*	Unknown	/
	***9L***	Interacts with STAT1 and STAT2 to induce degradation through apoptosis and the ubiquitin–proteasome pathways; Facilitates ASFV replication by degrading the host protein HAX1	[51,52]
	*10L*	Enhances the K48-linked ubiquitination of JAK1 by recruiting the E3 ubiquitin ligase HERC5	[25]
	*11L*	Facilitates the degradation of TBK1 and IRF7	[53]
	*12L*	Interferes with the interaction between p65 and importins, preventing nuclear localization	[54,55]
	*13L*	Facilitates the autophagic degradation of STING	[56]
	*14L*	Promotes IRF3 degradation by facilitating TRIM21-mediated K63-linked ubiquitination	[57]
	*15R*	Unknown	/
	*16R*	Interacts with the host proteins SERTAD3 and SDCBP, affecting viral replication and pathogenicity by regulating the transcriptional activity of host cell; Competes with BAX to bind HSP60, inducing apoptosis and affecting viral replication and pathogenicity by disrupting the HSP60-BAX complex	[58,59]
	*18R*	Unknown	/
	*19R*	Unknown	/
	*21R*	Unknown	/
*MGF505*	*1R*	Unknown	/
	*2R*	Interacts with STING	[60]
	*3R*	Facilitates TBK1 degradation through autophagy	[44]
	*4R*	Facilitates the autophagic degradation of TRAF3	[61]
	*5R*	Unknown	/
	*6R*	Facilitates degradation through the autophagy-lysosomal pathway and inhibits the K63-linked polyubiquitination of STING	[42]
	***7R***	Facilitates the autophagic degradation of STING; Inhibits IRF3 nuclear translocation; Suppresses the phosphorylation of I*κ*B*α*; Upregulates the expression of the E3 ubiquitin ligase RNF125, promoting the degradation of JAK1 and JAK2	[62,63,64,65]
	*9R*	Unknown	/
	*10R*	Unknown	/
	*11R*	Facilitates STING degradation through the lysosome, ubiquitin–proteasome, and autophagy pathways	[43]

Bold type indicates virulence-associated genes (VAGs). “/” represent the absence of references.

## Data Availability

Not applicable.

## References

[1] Wang T., Sun Y., Qiu H.J. (2018). African swine fever: An unprecedented disaster and challenge to China. Infect. Dis..

[2] Zhao D., Sun E., Huang L., Ding L., Zhu Y., Zhang J., Shen D., Zhang X., Zhang Z., Ren T. (2023). Highly lethal genotype I and II recombinant African swine fever viruses detected in pigs. Nat. Commun..

[3] Le V.P., Nguyen V.T., Le T.B., Mai N.T.A., Nguyen V.D., Than T.T., Lai T.N.H., Cho K.H., Hong S.K., Kim Y.H. (2024). Detection of recombinant African swine fever virus strains of p72 genotypes I and II in domestic pigs. Emerg. Infect. Dis..

[4] Chen S., Wang T., Luo R., Lu Z., Lan J., Sun Y., Fu Q., Qiu H.J. (2024). Genetic variations of African swine fever virus: Major challenges and prospects. Viruses.

[5] Dixon L.K., Chapman D.A., Netherton C.L., Upton C. (2013). African swine fever virus replication and genomics. Virus Res..

[6] Zsak L., Lu Z., Kutish G.F., Neilan J.G., Rock D.L. (1996). An African swine fever virus virulence-associated gene *NL-S* with similarity to the herpes simplex virus *ICP34.5* gene. J. Virol..

[7] Neilan J.G., Zsak L., Lu Z., Kutish G.F., Afonso C.L., Rock D.L. (2002). Novel swine virulence determinant in the left variable region of the African swine fever virus genome. J. Virol..

[8] Rathakrishnan A., Connell S., Petrovan V., Moffat K., Goatley L.C., Jabbar T., Sánchez-Cordón P.J., Reis A.L., Dixon L.K. (2022). Differential effect of deleting members of African swine fever virus *MGF360* and *MGF505* from the genotype II Georgia 2007/1 isolate on virus replication, virulence, and induction of protection. J. Virol..

[9] Afonso C.L., Piccone M.E., Zaffuto K.M., Neilan J., Kutish G.F., Lu Z., Balinsky C.A., Gibb T.R., Bean T.J., Zsak L. (2004). African swine fever virus *MGF360* and *MGF530* genes affect host interferon response. J. Virol..

[10] Blasco R., Agüero M., Almendral J.M., Viñuela E. (1989). Variable and constant regions in African swine fever virus DNA. Virology.

[11] Almendral J.M., Almazán F., Blasco R., Viñuela E. (1990). Multigene families in African swine fever virus: Family 110. J. Virol..

[12] González A., Calvo V., Almazán F., Almendral J.M., Ramírez J.C., De la Vega I., Blasco R., Viñuela E. (1990). Multigene families in African swine fever virus: Family 360. J. Virol..

[13] Vydelingum S., Baylis S.A., Bristow C., Smith G.L., Dixon L.K. (1993). Duplicated genes within the variable right end of the genome of a pathogenic isolate of African swine fever virus. J. Gen. Virol..

[14] Rodríguez J.M., Yañez R.J., Pan R., Rodríguez J.F., Salas M.L., Viñuela E. (1994). Multigene families in African swine fever virus: Family 505. J. Virol..

[15] Yozawa T., Kutish G.F., Afonso C.L., Lu Z., Rock D.L. (1994). Two novel multigene families, 530 and 300, in the terminal variable regions of African swine fever virus genome. Virology.

[16] Zhu Z., Chen H., Liu L., Cao Y., Jiang T., Zou Y., Peng Y. (2021). Classification and characterization of multigene family proteins of African swine fever viruses. Brief Bioinform..

[17] Dixon L.K., Islam M., Nash R., Reis A.L. (2019). African swine fever virus evasion of host defences. Virus Res..

[18] Dixon L.K., Sun H., Roberts H. (2019). African swine fever. Antivir. Res..

[19] Vu H.L.X., McVey D.S. (2024). Recent progress on gene-deleted live-attenuated African swine fever virus vaccines. NPJ Vaccines.

[20] Pires S., Ribeiro G., Costa J.V. (1997). Sequence and organization of the left multigene family 110 region of the Vero-adapted L60V strain of African swine fever virus. Virus Genes.

[21] Rodríguez J.M., Moreno L.T., Alejo A., Lacasta A., Rodríguez F., Salas M.L. (2015). Genome sequence of African swine fever virus BA71, the virulent parental strain of the nonpathogenic and tissue-culture adapted BA71V. PLoS ONE.

[22] Wang T., Wang L., Han Y., Pan L., Yang J., Sun M., Zhou P., Sun Y., Bi Y., Qiu H.J. (2021). Adaptation of African swine fever virus to HEK293T cells. Transbound. Emerg. Dis..

[23] Krug P.W., Holinka L.G., O’Donnell V., Reese B., Sanford B., Fernandez-Sainz I., Gladue D.P., Arzt J., Rodriguez L., Risatti G.R. (2015). The progressive adaptation of a Georgian isolate of African swine fever virus to Vero cells leads to a gradual attenuation of virulence in swine corresponding to major modifications of the viral genome. J. Virol..

[24] Li D., Liu Y., Qi X., Wen Y., Li P., Ma Z., Liu Y., Zheng H., Liu Z. (2021). African swine fever virus *MGF*-*110*-*9L*-deficient mutant has attenuated virulence in pigs. Virol. Sin..

[25] Li D., Peng J., Wu J., Yi J., Wu P., Qi X., Ren J., Peng G., Duan X., Ru Y. (2023). African swine fever virus *MGF*-360-*10L* is a novel and crucial virulence factor that mediates ubiquitination and degradation of JAK1 by recruiting the E3 ubiquitin ligase HERC5. mBio.

[26] Wang T., Luo R., Zhang J., Lan J., Lu Z., Zhai H., Li L.F., Sun Y., Qiu H.J. (2024). The African swine fever virus MGF300-4L protein is associated with viral pathogenicity by promoting the autophagic degradation of IKK*β* and increasing the stability of I*κ*B*α*. Emerg. Microbes Infect..

[27] Wang T., Luo R., Zhang J., Lu Z., Li L.F., Zheng Y.H., Pan L., Lan J., Zhai H., Huang S. (2023). The MGF300-2R protein of African swine fever virus is associated with viral pathogenicity by promoting the autophagic degradation of IKK*α* and IKK*β* through the recruitment of TOLLIP. PLoS Pathog..

[28] Farlow J., Donduashvili M., Kokhreidze M., Kotorashvili A., Vepkhvadze N.G., Kotaria N., Gulbani A. (2018). Intra-epidemic genome variation in highly pathogenic African swine fever virus from the country of Georgia. Virol. J..

[29] Mazur-Panasiuk N., Walczak M., Juszkiewicz M., Woźniakowski G. (2020). The spillover of African swine fever in western Poland revealed its estimated origin on the basis of *O174L*, *K145R*, *MGF 505*-*5R* and IGR *I73R*/*I329L* genomic sequences. Viruses.

[30] Mazloum A., Van Schalkwyk A., Shotin A., Igolkin A., Shevchenko I., Gruzdev K.N., Vlasova N. (2021). Comparative analysis of full genome sequences of African swine fever virus isolates taken from wild boars in Russia in 2019. Pathogens.

[31] Zhang Y. (2023). Tracing the origin of genotype II African swine fever virus in China by genomic epidemiology analysis. Transbound. Emerg. Dis..

[32] Thanh T.T., Duc T.A., Viet L.D., Van H.T., Thi N.C., Thi C.N., Thi N.H., Vu D.H. (2021). Rapid identification for serotyping of African swine fever virus based on the short fragment of the *EP402R* gene encoding for CD2-like protein. Acta Vet..

[33] Wang Z., Qi C., Ge S., Li J., Hu Y., Qian Y. (2022). Genetic variation and evolution of attenuated African swine fever virus strain isolated in the field: A review. Virus Res..

[34] Portugal R., Coelho J., Höper D., Little N.S., Smithson C., Upton C., Martins C., Leitão A., Keil G.M. (2015). Related strains of African swine fever virus with different virulence: Genome comparison and analysis. J. Gen. Virol..

[35] Sánchez E.G., Riera E., Nogal M., Gallardo C., Fernández P., Bello-Morales R., López-Guerrero J.A., Chitko-McKown C.G., Richt J.A., Revilla Y. (2017). Phenotyping and susceptibility of established porcine cells lines to African swine fever virus infection and viral production. Sci. Rep..

[36] Borca M.V., Rai A., Ramirez-Medina E., Silva E., Velazquez-Salinas L., Vuono E., Pruitt S., Espinoza N., Gladue D.P. (2021). A cell culture-adapted vaccine virus against the current African swine fever virus pandemic strain. J. Virol..

[37] Thaweerattanasinp T., Kaewborisuth C., Viriyakitkosol R., Saenboonrueng J., Wanitchang A., Tanwattana N., Sonthirod C., Sangsrakru D., Pootakham W., Tangphatsornruang S. (2024). Adaptation of African swine fever virus to MA-104 cells: Implications of unique genetic variations. Vet. Microbiol..

[38] Petrini S., Righi C., Mészáros I., D’Errico F., Tamás V., Pela M., Olasz F., Gallardo C., Fernandez-Pinero J., Göltl E. (2023). The production of recombinant African swine fever virus Lv17/WB/Rie1 strains and their in vitro and in vivo characterizations. Vaccines.

[39] Xu L., Lyu J., Qiu Z., Liu Q., Hu H., Zhao L., Pan M. (2025). Laminaran potentiates cGAS-STING signaling to enhance antiviral responses. Int. Immunopharmacol..

[40] Cheng W.Y., He X.B., Jia H.J., Chen G.H., Jin Q.W., Long Z.L., Jing Z.Z. (2018). The cGAS-STING signaling pathway is required for the innate immune response against ectromelia virus. Front. Immunol..

[41] Du Y., Hu Z., Luo Y., Wang H.Y., Yu X., Wang R.F. (2023). Function and regulation of cGAS-STING signaling in infectious diseases. Front. Immunol..

[42] Yao M., Cao H., Li W., Hu Z., Rong Z., Yin M., Tian L., Hu D., Li X., Qian P. (2024). African swine fever virus *MGF505*-*6R* attenuates type I interferon production by targeting STING for degradation. Front. Immunol..

[43] Yang K., Huang Q., Wang R., Zeng Y., Cheng M., Xue Y., Shi C., Ye L., Yang W., Jiang Y. (2023). Corrigendum to African swine fever virus *MGF505*-*11R* inhibits type I interferon production by negatively regulating the cGAS-STING-mediated signaling pathway. Vet. Microbiol..

[44] Cheng M., Luo J., Duan Y. (2022). African swine fever virus *MGF505*-*3R* inhibits cGAS-STING-mediated IFN-*β* pathway activation by degrading TBK1. Anim. Dis..

[45] Wang Z., He Y., Huang Y., Zhai W., Tao C., Chu Y., Pang Z., Zhu H., Zhao P., Jia H. (2024). African swine fever virus MGF360-4L protein attenuates type I interferon response by suppressing the phosphorylation of IRF3. Front. Immunol..

[46] Kim M.H., Subasinghe A., Kim Y., Kwon H.I., Cho Y., Chathuranga K., Cha J.W., Moon J.Y., Hong J.H., Kim J. (2024). Development and characterization of high-efficiency cell-adapted live attenuated vaccine candidate against African swine fever. Emerg. Microbes Infect..

[47] Zhong H., Fan S., Du Y., Zhang Y., Zhang A., Jiang D., Han S., Wan B., Zhang G. (2022). African swine fever virus *MGF110*-*7L* induces host cell translation suppression and stress granule formation by activating the PERK/PKR-eIF2*α* pathway. Microbiol. Spectr..

[48] Ren J., Li D., Zhu G., Yang W., Ru Y., Feng T., Qin X., Hao R., Duan X., Liu X. (2023). Deletion of *MGF*-*110*-*9L* gene from African swine fever virus weakens autophagic degradation of TBK1 as a mechanism for enhancing type I interferon production. FASEB J..

[49] Lu Z., Luo R., Lan J., Chen S., Qiu H.J., Wang T., Sun Y. (2024). The MGF300-2R protein of African swine fever virus promotes IKK*β* ubiquitination by recruiting the E3 ubiquitin ligase TRIM21. Viruses.

[50] Sun H., Yang J., Zhang Z., Wu M., Tian Z., Liu Y., Zhang X., Zhong J., Yang S., Chen Y. (2025). The African swine fever virus gene *MGF*_*360*-*4L* inhibits interferon signaling by recruiting mitochondrial selective autophagy receptor SQSTM1 degrading MDA5 antagonizing innate immune responses. mBio.

[51] Zhang K., Yang B., Shen C., Zhang T., Hao Y., Zhang D., Liu H., Shi X., Li G., Yang J. (2022). *MGF360*-*9L* is a major virulence factor associated with the African swine fever virus by antagonizing the JAK/STAT signaling pathway. mBio.

[52] Yang J., Yang B., Hao Y., Shi X., Yang X., Zhang D., Zhao D., Yan W., Chen L., Bie X. (2023). African swine fever virus *MGF360*-*9L* promotes viral replication by degrading the host protein HAX1. Virus Res..

[53] Yang K., Xue Y., Niu H., Shi C., Cheng M., Wang J., Zou B., Wang J., Niu T., Bao M. (2022). African swine fever virus *MGF360*-*11L* negatively regulates cGAS-STING-mediated inhibition of type I interferon production. Vet. Res..

[54] Zhuo Y., Guo Z., Ba T., Zhang C., He L., Zeng C., Dai H. (2021). African swine fever virus *MGF360*-*12L* inhibits type I interferon production by blocking the interaction of Importin *α* and NF-*κ*B signaling pathway. Virol. Sin..

[55] Chen Q., Wang X.X., Jiang S.W., Gao X.T., Huang S.Y., Liang Y., Jia H., Zhu H.F. (2023). *MGF360*-*12L* of ASFV-SY18 is an immune-evasion protein that inhibits host type I IFN, NF-*κ*B, and JAK/STAT pathways. Pol. J. Vet. Sci..

[56] Luo J., Cheng M., Duan Y., Xing X., Lu M., Sun Y., Shi C., Wang J., Lu Y., Li X. (2023). African swine fever virus encoded protein *MGF360*-*13L* inhibits cGAS-STING-mediated IFN-I signaling pathway. Gene.

[57] Wang Y., Cui S., Xin T., Wang X., Yu H., Chen S., Jiang Y., Gao X., Jiang Y., Guo X. (2022). African swine fever virus *MGF360*-*14L* negatively regulates type I interferon signaling by targeting IRF3. Front. Cell Infect. Microbiol..

[58] Ramirez-Medina E., Vuono E.A., Velazquez-Salinas L., Silva E., Rai A., Pruitt S., Berggren K.A., Zhu J., Borca M.V., Gladue D.P. (2020). The *MGF360*-*16R* ORF of African swine fever virus strain Georgia encodes for a nonessential gene that interacts with host proteins SERTAD3 and SDCBP. Viruses.

[59] Xiang Z., Xu Z., Weng W., Wang H., Wu J., Jiang F., Qu Y., Li Q., Gao P., Zhou L. (2025). The African swine fever virus *MGF360-16R* protein functions as a mitochondrial-dependent apoptosis inducer by competing with BAX to bind to the HSP60 protein. J. Virol..

[60] Sunwoo S.Y., García-Belmonte R., Walczak M., Vigara-Astillero G., Kim D.M., Szymankiewicz K., Kochanowski M., Liu L., Tark D., Podgórska K. (2024). Deletion of *MGF505*-*2R* gene activates the cGAS-STING pathway leading to attenuation and protection against virulent African swine fever virus. Vaccines.

[61] Dupré J., Le Dimna M., Hutet E., Dujardin P., Fablet A., Leroy A., Fleurot I., Karadjian G., Roesch F., Caballero I. (2024). Exploring type I interferon pathway: Virulent vs. attenuated strain of African swine fever virus revealing a novel function carried by *MGF505-4R*. Front. Immunol..

[62] Ding M., Dang W., Liu H., Xu F., Huang H., Sun Y., Li T., Pei J., Liu X., Zhang Y. (2022). Combinational deletions of *MGF360*-*9L* and *MGF505*-*7R* attenuated highly virulent African swine fever virus and conferred protection against homologous challenge. J. Virol..

[63] Li J., Song J., Kang L., Huang L., Zhou S., Hu L., Zheng J., Li C., Zhang X., He X. (2021). *pMGF505-7R* determines pathogenicity of African swine fever virus infection by inhibiting IL-1*β* and type I IFN production. PLoS Pathog..

[64] Huang L., Li J., Zheng J., Li D., Weng C. (2022). Multifunctional *pMGF505-7R* is a key virulence-related factor of African swine fever virus. Front. Microbiol..

[65] Huang Z., Cao H., Zeng F., Lin S., Chen J., Luo Y., You J., Kong C., Mai Z., Deng J. (2023). African swine fever virus *MGF505*-*7R* interacts with interferon regulatory factor 9 to evade the type I interferon signaling pathway and promote viral replication. J. Virol..

[66] Villarino A.V., Kanno Y., Ferdinand J.R., O’Shea J.J. (2015). Mechanisms of JAK/STAT signaling in immunity and disease. J. Immunol..

[67] Nishio H., Matsui K., Tsuji H., Tamura A., Suzuki K. (2001). Immunolocalisation of the Janus kinases (JAK)-signal transducers and activators of transcription (STAT) pathway in human epidermis. J. Anat..

[68] O’Donnell V., Holinka L.G., Gladue D.P., Sanford B., Krug P.W., Lu X., Arzt J., Reese B., Carrillo C., Risatti G.R. (2015). African swine fever virus Georgia isolate harboring deletions of *MGF360* and *MGF505* genes is attenuated in swine and confers protection against challenge with virulent parental virus. J. Virol..

[69] Chen W., Zhao D., He X., Liu R., Wang Z., Zhang X., Li F., Shan D., Chen H., Zhang J. (2020). A seven-gene-deleted African swine fever virus is safe and effective as a live attenuated vaccine in pigs. Sci. China Life Sci..

[70] Reis A.L., Abrams C.C., Goatley L.C., Netherton C., Chapman D.G., Sanchez-Cordon P., Dixon L.K. (2016). Deletion of African swine fever virus interferon inhibitors from the genome of a virulent isolate reduces virulence in domestic pigs and induces a protective response. Vaccine.

[71] Li D., Ren J., Zhu G., Wu P., Yang W., Ru Y., Feng T., Liu H., Zhang J., Peng J. (2023). Deletions of *MGF110*-*9L* and *MGF360*-*9L* from African swine fever virus are highly attenuated in swine and confer protection against homologous challenge. J. Biol. Chem..

[72] Li D., Yang W., Li L., Li P., Ma Z., Zhang J., Qi X., Ren J., Ru Y., Niu Q. (2021). African swine fever virus MGF-505-7R negatively regulates cGAS–STING-mediated signaling pathway. J. Immunol..

[73] Liu Y., Li Y., Xie Z., Ao Q., Di D., Yu W., Lv L., Zhong Q., Song Y., Liao X. (2021). Development and in vivo evaluation of *MGF100-1R* deletion mutant in an African swine fever virus Chinese strain. Vet. Microbiol..

[74] Ramirez-Medina E., Vuono E., Pruitt S., Rai A., Silva E., Espinoza N., Zhu J., Velazquez-Salinas L., Borca M.V., Gladue D.P. (2021). Development and in vivo evaluation of a MGF110-1L deletion mutant in African swine fever strain Georgia. Viruses.

[75] Ramirez-Medina E., Vuono E., Silva E., Rai A., Valladares A., Pruitt S., Espinoza N., Velazquez-Salinas L., Borca M.V., Gladue D.P. (2022). Evaluation of the deletion of *MGF110-5L-6L* on swine virulence from the pandemic strain of African swine fever virus and use as a DIVA marker in vaccine candidate ASFV-G-ΔI177L. J. Virol..

[76] Tamás V., Righi C., Mészáros I., D’Errico F., Olasz F., Casciari C., Zádori Z., Magyar T., Petrini S., Feliziani F. (2023). Involvement of the *MGF 110-11L* gene in the African swine fever replication and virulence. Vaccines.

[77] Ramirez-Medina E., Vuono E.A., Rai A., Pruitt S., Silva E., Velazquez-Salinas L., Zhu J., Gladue D.P., Borca M.V. (2020). Evaluation in swine of a recombinant African swine fever virus lacking the *MGF-360-1L* gene. Viruses.

[78] Reis A.L., Goatley L.C., Jabbar T., Sanchez-Cordon P.J., Netherton C.L., Chapman D.A.G., Dixon L.K. (2017). Deletion of the African swine fever virus gene DP148R does not reduce virus replication in culture but reduces virus virulence in pigs and induces high levels of protection against challenge. J. Virol..

[79] Zsak L., Lu Z., Burrage T.G., Neilan J.G., Kutish G.F., Moore D.M., Rock D.L. (2001). African swine fever virus multigene family 360 and 530 genes are novel macrophage host range determinants. J. Virol..

[80] Burrage T.G. (2013). African swine fever virus infection in *Ornithodoros* ticks. Virus Res..

[81] Burrage T.G., Lu Z., Neilan J.G., Rock D.L., Zsak L. (2004). African swine fever virus *MGF360* genes affect virus replication and generalization of infection in *Ornithodoros porcinus* ticks. J. Virol..

[82] Hu Z., Tian X., Lai R., Wang X., Li X. (2023). Current detection methods of African swine fever virus. Front. Vet. Sci..

[83] Zhao K., Shi K., Zhou Q., Xiong C., Mo S., Zhou H., Long F., Wei H., Hu L., Mo M. (2022). The development of a multiplex real-time quantitative PCR assay for the differential detection of the wild-type strain and the *MGF505*-*2R*, *EP402R* and *I177L* gene-deleted strain of the African swine fever virus. Animals.

[84] Zuo X., Peng G., Xia Y., Xu L., Zhao Q., Zhu Y., Wang C., Liu Y., Zhao J., Wang H. (2023). A quadruple fluorescence quantitative PCR method for the identification of wild strains of African swine fever and gene-deficient strains. Virol. J..

[85] Wang L., Li Y., Zhang X., Madera R., Pantua H., Craig A., Muro N., Li D., Retallick J., Ferreyra F.M. (2025). Specific detection of African swine fever virus variants: Novel quadplex real-time PCR assay with internal control. Microorganisms.

[86] Yang H., Peng Z., Song W., Zhang C., Fan J., Chen H., Hua L., Pei J., Tang X., Chen H. (2022). A triplex real-time PCR method to detect African swine fever virus gene-deleted and wild type strains. Front. Vet. Sci..

[87] Velazquez-Salinas L., Ramirez-Medina E., Rai A., Pruitt S., Vuono E.A., Espinoza N., Gladue D.P., Borca M.V. (2021). Development real-time PCR assays to genetically differentiate vaccinated pigs from infected pigs with the Eurasian strain of African swine fever virus. Front. Vet. Sci..

[88] Guo Z., Li K., Qiao S., Chen X.X., Deng R., Zhang G. (2020). Development and evaluation of duplex TaqMan real-time PCR assay for detection and differentiation of wide-type and *MGF505*-*2R* gene-deleted African swine fever viruses. BMC Vet. Res..

[89] Qi C., Zhang Y., Wang Z., Li J., Hu Y., Li L., Ge S., Wang Q., Wang Y., Wu X. (2023). Development and application of a TaqMan-based real-time PCR method for the detection of the ASFV *MGF505*-*7R* gene. Front. Vet. Sci..

[90] Shi K., Zhao K., Wei H., Zhou Q., Shi Y., Mo S., Long F., Hu L., Feng S., Mo M. (2023). Triplex crystal digital PCR for the detection and differentiation of the wild-type strain and the *MGF505*-*2R* and *I177L* gene-deleted strain of African swine fever virus. Pathogens.

[91] Hu Z., Lai R., Tian X., Guan R., Li X. (2024). A duplex fluorescent quantitative PCR assay to distinguish the genotype I, II and I/II recombinant strains of African swine fever virus in China. Front. Vet. Sci..

[92] O’Donnell V., Holinka L.G., Sanford B., Krug P.W., Carlson J., Pacheco J.M., Reese B., Risatti G.R., Gladue D.P., Borca M.V. (2016). African swine fever virus Georgia isolate harboring deletions of *9GL* and *MGF360/505* genes is highly attenuated in swine but does not confer protection against parental virus challenge. Virus Res..

[93] Liu Y., Xie Z., Li Y., Song Y., Di D., Liu J., Gong L., Chen Z., Wu J., Ye Z. (2023). Evaluation of an *I177L* gene-based five-gene-deleted African swine fever virus as a live attenuated vaccine in pigs. Emerg. Microbes Infect..

[94] Gong W., Li J., Wang Z., Sun J., Mi S., Xu J., Cao J., Hou Y., Wang D., Huo X. (2019). Commercial E2 subunit vaccine provides full protection to pigs against lethal challenge with 4 strains of classical swine fever virus genotype 2. Vet. Microbiol..

[95] Guo J., Hou L., Zhou J., Wang D., Cui Y., Feng X., Liu J. (2022). Porcine circovirus type 2 vaccines: Commercial application and research advances. Viruses.

[96] Wang H., Shan S., Wang S., Zhang H., Ma L., Hu L., Huang H., Wei K., Zhu R. (2017). Fused IgY Fc and polysaccharide adjuvant enhanced the immune effect of the recombinant VP2 and VP5 subunits-a prospect for improvement of infectious bursal disease virus subunit vaccine. Front. Microbiol..

[97] Bosch-Camós L., López E., Collado J., Navas M.J., Blanco-Fuertes M., Pina-Pedrero S., Accensi F., Salas M.L., Mundt E., Nikolin V. (2021). M448R and MGF505-7R: Two African swine fever virus antigens commonly recognized by ASFV-specific T-cells and with protective potential. Vaccines.

[98] Netherton C.L., Goatley L.C., Reis A.L., Portugal R., Nash R.H., Morgan S.B., Gault L., Nieto R., Norlin V., Gallardo C. (2019). Identification and immunogenicity of African swine fever virus antigens. Front. Immunol..

[99] Goatley L.C., Reis A.L., Portugal R., Goldswain H., Shimmon G.L., Hargreaves Z., Ho C.S., Montoya M., Sánchez-Cordón P.J., Taylor G. (2020). A pool of eight virally vectored African swine fever antigens protect pigs against fatal disease. Vaccines.

